# Assessment of growth performance of striped catfish (*Pangasianodon hypophthalmus*) and yield of Guinea grass (*Panicum maximum* cv. Mombaça) under a biosaline integrated aquaculture-agriculture system

**DOI:** 10.1186/s12870-025-07401-0

**Published:** 2025-09-22

**Authors:** Khaled Madkour, Fahad Kimera, Muziri Mugwanya, Mahmoud A.O. Dawood, Hani Sewilam

**Affiliations:** 1 Researcher, Center for Applied Research on the Environment and Sustainability (CARES), School of Science and Engineering, The American University in Cairo, PO Box 74, AUC Avenue, New Cairo, 11835 Egypt; 2 Senior Researcher, Center for Applied Research on the Environment and Sustainability (CARES), School of Science and Engineering, The American University in Cairo, P.O. Box 74, AUC Avenue, New Cairo, 11835 Egypt; 3 Researcher, Center for Applied Research on the Environment and Sustainability (CARES), School of Science and Engineering, The American University in Cairo, P.O. Box 74, AUC Avenue, New Cairo, 11835 Egypt; 4 Center for Applied Research on the Environment and Sustainability (CARES), School of Science and Engineering, The American University in Cairo, P.O. Box 74, AUC Avenue, New Cairo, 11835 Egypt; 5 Animal Production Department, Faculty of Agriculture, Kafrelsheikh University, Kafr El- Sheikh, Kafr El-Shaikh, Egypt; 6 Managing Director, UNESCO Chair in Hydrological Changes and Water Resources Management, RWTH Aachen University, Aachen, Germany; 7 Center for Applied Research on the Environment and Sustainability (CARES), School of Science and Engineering, The American University in Cairo, P.O. Box 74, AUC Avenue, New Cairo, 11835 Egypt

**Keywords:** Animal feed, Brackish water, Forage quality, Salinity, Sustainability

## Abstract

**Background:**

With freshwater scarcity and soil infertility being major challenges farmers face in arid and semi-arid regions, a biosaline-integrated aquaculture-agriculture system (IAAS) is key to diversifying yields with limited inputs in marginal areas. This research investigated the effect of varying brackish water salinities on the growth and yield of striped catfish (*Pangasianodon hypophthalmus*) and Guinea grass (*Panicum maximum* cv. Mombaça) in a biosaline-IAAS. The study followed a randomized completely block design under three replications of three aquaculture effluent salinity treatments and freshwater control, namely: 500 (control), 5000, 10,000, and 15,000 mg/L (i.e., CT, SM5, SM10, and SM15, respectively). Morphological plant parameters, forage quality and fish growth performance were evaluated. The plants were harvested in 3 consecutive cuts during the seven-month study period.

**Results:**

The study results showed that the canopy height, leaf number, and tiller number decreased with the increasing number of cuts and salinity. At cut 3, the control significantly recorded higher values for canopy height, chlorophyll content, and tiller number per plant compared with other salinity treatments. Yield data showed no significant differences in stalk fresh and dry weights among the treatments at cuts 1 and 2, except for the control at cut 3. Results on forage quality parameters, such as neutral detergent fiber, acid detergent fiber, and acid detergent lignin, showed an increasing trend with the increasing number of cuts. Furthermore, the results also showed that plants irrigated with saline fish effluents exhibited lower values for crude protein compared with the control. Regarding in vitro digestibility, results revealed a general decrease in the in vitro true digestibility and digestible organic Matter percentages among salinity treatments with increasing cut numbers. For striped catfish, growth performance was affected when reared in water salinities exceeding 10,000 mg/L.

**Conclusion:**

The integration of striped catfish and Mombaca at water salinities not exceeding 10,000 mg/L could be a feasible alternative in diversifying food and feed production in marginal areas.

**Supplementary Information:**

The online version contains supplementary material available at 10.1186/s12870-025-07401-0.

## Background

Land salinization is among the major threats hindering current developments in irrigated agriculture [1, 2]. Over time, deterioration in underground water quality and the cumulative build-up of salt concentrations in the top agricultural soil layers have consequently led to decreased crop yields, limited crop diversity, and accelerated soil degradation [3–5]. This effect is because most plants are susceptible to salinity, and the number of lands impacted by this effect is significantly increasing [6–8]. In the last two decades, there has been an increase in saline irrigation across the globe, especially in the Mediterranean regions, due to freshwater scarcity, thus leading to reduced crop yields and diversification, which has threatened food security [9–11]. Moreover, the excessive use of chemical fertilizers has magnified the salinity effect, further worsening land degradation [12, 13]. Therefore, it is important to search for sustainable agricultural practices aimed at improving soil quality and crop productivity under saline conditions in marginal areas.

Biosaline-integrated aquaculture-agriculture systems (IAAS) have been proposed as suitable alternatives for increasing brackish water productivity and yield diversification in marginal areas with limited inputs while minimizing the environmental impact [14–16]. An integrated aquaculture-agriculture system is a method of production where two or more aquaculture and agricultural operations operate simultaneously or in succession [17, 18]. As such, wastes from one system are recycled as inputs for another, thus reducing environmental pollution [19, 20]. The pollution resulting from the disposal of fish effluents in the environment causes the build-up of organic matter in aquatic ecosystems, which results in eutrophication [21–23]. Hence, reusing fish effluents in IAAS improves the farm output [24]. IAAS can be implemented using systems that combine fish and livestock production, fish and poultry production, or fish and crop production [25–27]. The integration of fish and crop production necessitates that the farmer has prior knowledge of the optimal conditions required by both the fish and crops for proper growth and survival under different environmental conditions [28]. This study used striped catfish (*Pangasianodon hypophthalmus***)** and Guinea grass (*Panicum maximum* cv. Mombaça) as model organisms for biosaline-IAAS.

Striped catfish exhibits better feed intake (FI), feed conversion ratio (FCR), and growth when reared in salinity concentrations between 2000 and 10,000 mg/L. However, these fish can still survive in water salinities reaching up to 18,000 mg/L, above which they show growth retardation, deformities and death [29, 30]. On the other hand, Guinea grass (*Panicum maximum*), a widely distributed forage crop in the tropics, has been reported to have greater adaptability to different ecological zones, better yields, and higher nutritional value compared to other grass/forage species in the Poaceae family [31]. It is one of the most significant fodder crops and an important commercial forage plant worldwide [16]. Guinea grass is a crop with the potential for dry Matter yield, with annual yields of up to 11.94 t DMY/ha in subtropical and tropical areas [31]. When compared to the other four fodder species, such as *Euchlaena mexicana*, *Brachiaria brizantha*, *Setaria sphacelata*, and *Cynodon plectostachyus*, Guinea grass exhibited better tolerance to salinity levels reaching up to 8,800 mg/L (EC = 11 dS/m), followed by *S. sphacelata* and *E. mexicana*, respectively [32]. The cultivar Mombaça from Brazil is the most commercialized and cultivated genotype of grass in North African countries. The adaptability of this cultivar in dry and semi-arid conditions is the reason for its spread in the climates of North African countries, where drought, salinity, and winter stress are all present [31]. Mombaca uses different mechanisms to overcome salinity stress, such as using salt-secreting micro hairs to expel the absorbed salts, keeping salts away from the photosynthetic area, accumulation of osmolytes in tissues, as well as senescence of the old leaves to ensure the survival and growth of the next generation of tillers under salinity conditions [33, 34]. Therefore, Mombaca can be a substitute for traditional forage crops in marginal regions [35].

This current research aimed to study the effect of different water salinities on the morphological growth, yield, and forage quality of Mombaça and the growth performance of striped catfish under a biosaline-IAAS. We hypothesize that the integration of growing Mombaca using the fish effluent of striped catfish at different water salinities can be a feasible way to optimize brackish water productivity in arid and semi-arid environments.

## Materials and methods

**Fig. 1:**
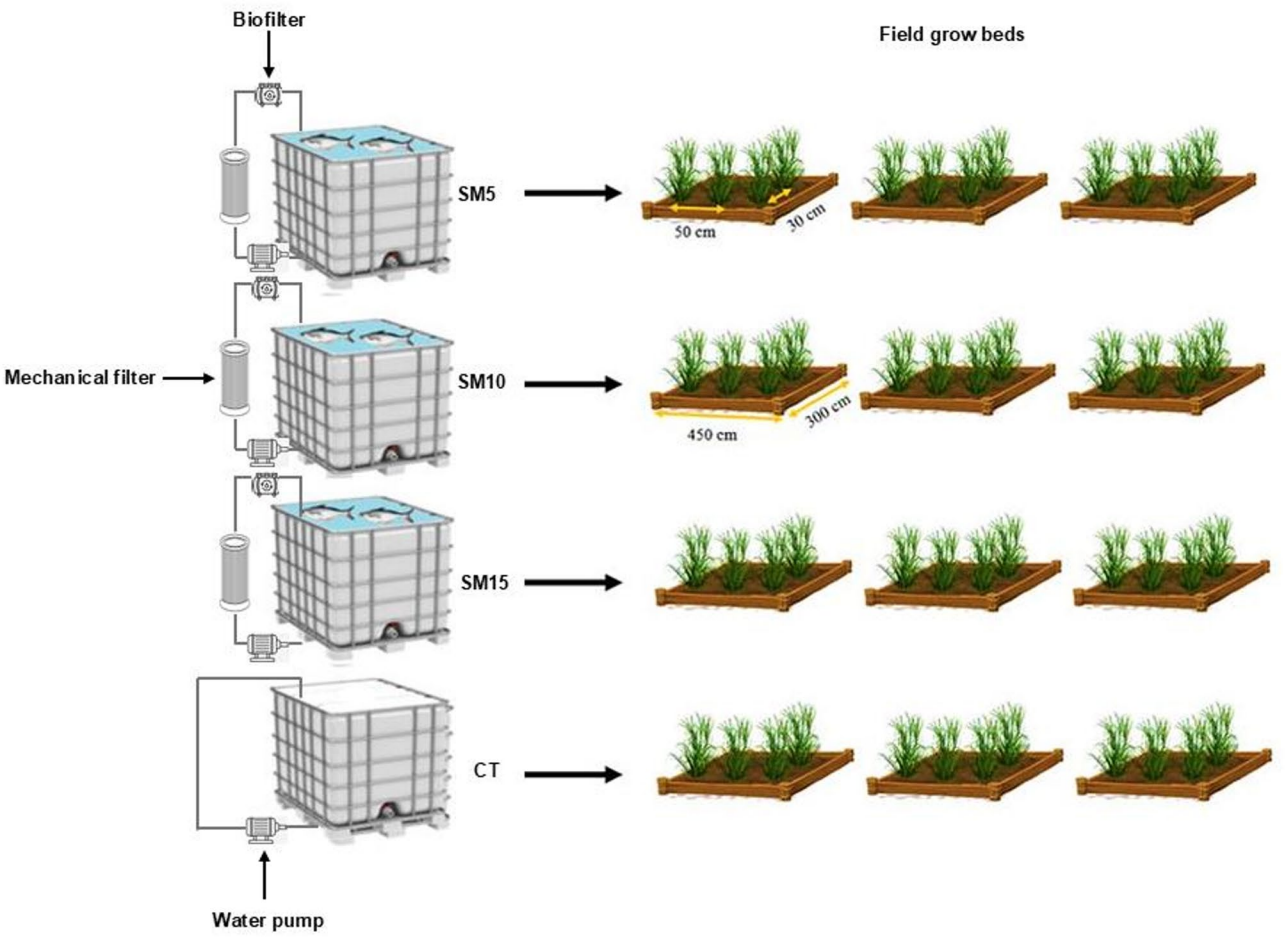
Schematic illustration of the experimental design. Fish were reared in different water salinity levels, which correspond to different irrigation treatments in experimental crop plots: CT (control), SM5 (5000 mg/L), SM10 (10,000 mg/L), and SM15 (15,000 mg/L)

A field experiment was conducted for seven months between July 10th and October 11th, 2021, at the Center for Applied Research on the Environment and Sustainability at the American University in Cairo in New Cairo, Egypt (30°01′11.7′′N 31°29′59.8′′E). Throughout the growing season, the minimum and Maximum temperatures were 6.9 °C and 36.2 °C, with an average temperature of 20.53 °C. The average precipitation and solar radiation were 4 mm and 220.2 W/m^2^, respectively. The experiment was laid out in a randomized completely block design (RCBD) with three salinity treatments and a control, under three replicates per treatment. The treatments were tested in experimental plots measuring 4.5 m by 3 m with an intra and inter-row spacing of 20 cm and 60 cm, respectively. The experimental layout was composed of the following irrigation treatments: (1) fish effluent with a salinity of 5000 mg/L (SM5), (2) fish effluent with a salinity of 10,000 mg/L (SM10), (3) fish effluent with a salinity of 15,000 mg/L (SM15), and (4) freshwater mixed with chemical fertilizers as a control treatment, 500 mg/L (CT) as presented in Fig. 1.

### Plants and soil materials

Thirty-day-old seedlings of *Panicum maximum* cv. Mombaca (Super Mombaça F1), from now on referred to as Mombaca, was sourced from the Agricultural Research Center in Dokki, Cairo, Egypt. The seedlings were transplanted in May 2021 in experimental plots and irrigated with respective water quality and nutrition treatments. Chemical fertilizers were applied at the recommended rates of 300 kg/ha nitrogen and 70 kg/ha phosphorus(P_2_O_5_) [36]. The sand in the grow beds was obtained from Fayoum and washed with freshwater before the implementation of the experimental project to eliminate dust that would affect the turbidity of water where fish are cultured. The synthetic salt used in the study was procured from the El-Nasr Salines company in Cairo, Egypt, and its chemical characteristics are shown in Table 1. Likewise, the physical and chemical parameters of the sand used in this study are presented in Table 2.

**Table 1:** Chemical properties of the salt used in this study

Element	Concentration
Sodium chloride	98.50%
Soluble matter	0.57%
Magnesium	0.07%
Calcium	0.07%
Potassium	0.02%
Sulfate	0.31%
Insoluble matter	0.02%
Moisture content	0.23%
Bicarbonate	4 × 10^−3^%
Copper	2 × 10^−6^%
Iron	3 × 10^−6^%
Mercury	5 × 10^−6^%
Lead	2 × 10^−5^%
Arsenate	2 × 10^−5^%
Cadmium	8 × 10^−7^%

**Table 2:** Physical and chemical characteristics of the sand used in the study

			Anions (meq/L)				Cations (meq/L)			
pH	EC (ppm)	SP	CO_3_^_^	HCO_3_^_^	Cl^−^	SO_4_^_^	Ca^++^	Mg^++^	Na^+^	K^+^
7.6	1376	23.00	-	2.36	8.47	11.61	10.61	6.28	5.13	0.43
Available macro and micronutrients (mg/kg)										
			N	P	K	Mn	Zn	Fe	Cu	
			47.00	14.54	48.00	0.43	0.16	1.44	0.06	

*EC* electroconductivity, *SP* sand particle size distribution, anions, CO_3_ carbonate, HCO_3_ hydrogen carbonate, *Cl* chloride, SO_4_^2^ sulfate, cations, Ca^++^ calcium, Mg^++^ magnesium, Na^+^ sodium, and K^+^ potassium

A field-scout TDR 350 soil moisture meter (Spectrum Technologies, Illinois, USA) was used to measure the volumetric water content of the soil (%). As such, all the plots were irrigated daily in the summer and every other day at the rate of 8 L/m^2^/day to maintain the field capacity. All the plants in the various water treatments were harvested/cut three times every 30 days after sowing. The herbage cuts were done 20 cm above the soil level to avoid causing damage to the plant stand and upcoming sampling operations [37].

### Plant growth measurements

Six planting spots (5 plants per spot) from each treatment per replicate within the borders were tagged for sampling. Plant height (cm), number of leaves, number of tillers per plant, number of dead leaves, leaf areas, fresh and dry weight of the leaves and stalks, and chlorophyll content (SPAD) were taken every 15 days. Plant height was measured using a meter rule from the base of the plant on the soil surface to the terminal growing point, leaf number was obtained by counting the number of fully expanded healthy leaves per tiller and determining averages; tiller number was obtained by counting the number of tillers per plant and determining averages, and chlorophyll content was measured using an MC-100 chlorophyll meter from Apogee Instruments, Inc., Utah, USA, in the early morning hours at each cutting time interval. Data were reported as SPAD means.

### The fish production units

Tilapia fingerlings were sourced from a private farm in Kafr El-Sheikh town in Egypt, sorted, and uniformly cultured in 1 m^3^ plastic Tanks under a fan and pad cooling greenhouse structure; each Tank was stocked with 100 individual fish of similar average initial weight of 6.0 g ± 0.25. Fish were fed three times per day until satiation (2–3% of their body biomass) on commercial pellets containing 30% proteins, 5% crude lipid, 6% crude fiber, 13% Ash, and 9% moisture content supplied by Skretting Egypt. Each Tank was supported with continuous aeration, and the discharged water for irrigation from each Tank was compensated when the water tank level reached 50%. The tanks were initially filled with dechlorinated tap water; then, the salinity was increased gradually by mixing salt to adapt the fish to the tested salinities [38]. According to the ammonia level (> 1 mg/L), 1% of the rearing water was replaced once or twice a week to keep the ammonia concentrations below 1 mg/L. The dissolved oxygen in the Tanks was in the range of 7.5–8 mg/L and was measured using a hand-held digital oxygen meter from Thermo Scientific (Orion Star™ A223). The fish were fed commercial pellets provided by the Skretting Egypt factory (30% crude protein) twice a day until satiation. Nutrient-rich fish effluent water, containing dissolved nutrients, fish faecal matter, and unconsumed fish feeds, was pumped through irrigation pipes using a submersible pump to direct water to irrigate the crops under a drip system. Drip irrigation lines were cleansed periodically to unclog the blocked drippers, aiming to increase the efficiency of the irrigation system.

### Fish wastewater quality

Automated digital Nilebot technologies by Conative Labs, Egypt, were utilized to closely monitor water quality parameters, such as water temperature, pH, and dissolved oxygen (DO), to ensure that they were within the optimum range for freshwater species (i.e. water temperature 24–27 °C, pH 6.8–7.8, and dissolved oxygen 5–8 mg/L). Water analysis chemical kits from HANNA Instruments were used to measure the nitrogenous elements in rearing water once every two weeks till the end of the experimental study. Briefly, 100 ml of rearing water was taken from each tank and immediately taken to the lab for analysis. HANNA reagent kits for nitrite (H193707-01), nitrate (H193728-01), and ammonia (H193715-01) were used to measure the nitrite, nitrate, and ammonia levels in water samples using an aquaculture photometer device (H18330). The instrument was programmed to show the nitrite-nitrogen, nitrate-nitrogen, ammonia-nitrogen, and ammonium concentrations in mg/L. The real-time analysis results obtained from the photometer were reported as mean values.

### Fish sampling and measurements

At the end of the trial, all fish were starved for 24 h before the final sampling. Then, the fish were weighed in bulk and counted to calculate the growth performance and survival rate (SR). The feed intake was recorded during the trial and used to calculate the feed conversion ratio (FCR). The formulae below were used to compute the following fish growth variables: feed conversion ratio (FCR), specific growth rate (SGR), feed intake (FI), body weight gain (BWG), and survival rate (SR%).

- BWG = Final body weight − Initial body weight.
- FCR = FI/BWG.
- SGR = (ln (Final body weight) − ln (Initial body weight))/Number of days.
- SR = (Number of fish at the end of the study/Number of fish at the beginning of the experiment) × 100.

Blood biochemical parameters were estimated as previously described by Mugwanya et al. [14], and the results are presented in Supplementary Tables 1 and Supplementary Table 2.

### Forage quality

For forage quality analysis, 5 plants per replicate in each treatment were pooled, and samples were taken to the Food and Feed Analysis lab of the Agricultural Research Center, Giza, Egypt, for processing and analysis. Fiber fractions analysis included Neutral detergent fiber (NDF), Acid detergent fiber (ADF), and Acid detergent lignin (ADL). The semiautomatic ANKOM220 Fiber Analyzer (ANKOM Technology, Macedon, NY, USA) was used to determine acid detergent lignin (ADL, AOAC no. 973.18), neutral detergent fiber (NDF, AOAC no. 2002.04), and acid detergent fiber (ADF, AOAC no. 973.18) as previously reported by Kimera et al. [15]. The organic matter of the detergent fiber fractions was estimated to yield quantities of cellulose (ADF-ADL), hemicellulose (NDF-ADF), and lignin. Total Digestible Nutrients (TDN) were calculated following the methods outlined by Atis et al. [39]

In-vitro digestible dry matter (IVTD DM%) was estimated as designated by Menke& Steingass [40]. The microbial protein was evaluated from the equation MP (g/kg DOM) = 120 X DOM/100 as defined by Czerkawski [41]. Short-chain fatty acids (SCFA) were estimated using the following formula: SCFA (mmol/ml gas) = (0.0239 x GP + 0.0601) as defined by Gelder et al. [42].

### Statistical analysis

Statistical analysis was performed on the data using IBM SPSS statistical software (version 22). Datasets were subjected to normality and equality of variance tests using Q-Q plots and Levene’s test, respectively. The data were analyzed by an analysis of variance (ANOVA), and the difference of means was conducted by the Tukey HSD test (*p* < 0.05).

## Results

### Growth parameters

The figures for the growth parameters of Mombaca, including plant height, leaf number per plant, SPAD value, tiller number, number of dead leaves, and leaf area, are presented in Table 3. For cut 1, there was a variation in the measured plant parameters as affected by different treatments, with SM15 recording higher values for leaf number, SPAD, and number of dead leaves. However, the effect of salinity was more pronounced in cut 2 and cut 3, where SM15 recorded the lowest values compared to other treatments. Overall, there was a significant interaction between cutting time points and treatments for all the measured plant parameters. However, no significant interaction was noted between blocks and treatments.

**Table 3:** Morphological plant parameters of *Panicum maximum* cv. Mombaça cultivated under different salinity treatments and at different cuts

Cut 1 (30 DAS)						
Treatments	Plant height (cm)	Leaf No./plant	SPAD	Tiller number/plant	No. of dead leaves/plant	Leaf Area (cm ^2^ )
CT	126.06^a^ ± 4.74	74.73^b^ ± 11.25	32.90^a^ ± 1.01	49.53^b^ ± 8.04	5.33^b^ ± 0.27	133.73^b^ ± 4.25
SM5	111.88^a^ ± 3.46	99.22^ab^ ± 13.72	32.40^a^ ± 0.55	81.89^a^ ± 14.17	8.11^a^ ± 0.72	137.95^b^ ± 3.88
SM10	119.50^a^ ± 5.31	100.85^ab^ ± 12.57	30.87^a^ ± 1.22	60.00^ab^ ± 8.04	8.07^a^ ± 0.91	158.25^a^ ± 6.16
SM15	125.00^a^ ± 3.45	116.55^a^ ± 7.95	35.23^a^ ± 1.39	54.56^ab^ ± 10.22	9.11^a^ ± 0.90	155.33^a^ ± 8.09
Cut 2 (60 DAS)						
CT	138.36^a^ ± 4.43	92.56^a^ ± 6.86	44.17^a^ ± 1.05	37.87^a^ ± 2.62	11.75^a^ ± 0.96	162.74^b^ ± 5.2
SM5	139.60^a^ ± 4.03	91.17^a^ ± 7.2	33.11^b^ ± 1.20	34.43^ab^ ± 3.28	9.57^a^ ± 0.84	195.16^a^ ± 7.22
SM10	136.76^a^ ± 3.15	87.93^a^ ± 5.86	34.85^b^ ± 0.79	32.47^ab^ ± 1.76	9.20^a^ ± 0.87	176.50^b^ ± 5.29
SM15	122.17^b^ ± 4.75	73.00^b^ ± 6.29	32.78^b^ ± 0.79	29.00^b^ ± 2.61	10.78^a^ ± 0.99	138.78^c^ ± 5.5
Cut 3 (90 DAS)						
CT	149.70^a^ ± 3.08	105.26^a^ ± 9.70	57.25^a^ ± 2.20	40.67^a^ ± 4.91	14.60^a^ ± 0.94	115.09^a^ ± 3.94
SM5	115.18^b^ ± 4.08	75.27^ab^ ± 11.85	33.05^b^ ± 2.35	25.73^b^ ± 5.01	13.27^a^ ± 1.15	113.08^a^ ± 4.71
SM10	113.99^b^ ± 5.11	89.42^a^ ± 10.71	35.51^b^ ± 2.24	24.07^b^ ± 3.13	13.71^a^ ± 0.82	108.36^a^ ± 4.38
SM15	108.57^b^ ± 3.91	46.22^b^ ± 7.19	33.85^b^ ± 1.49	20.55^b^ ± 2.50	12.67^a^ ± 2.00	102.69^a^ ± 4.26
DAS*Treatments	***	***	***	**	*	ns
Blocks*Treatments	ns	ns	ns	ns	ns	ns

Data is expressed as mean ± SD. Different lower superscript letters within each column per cut indicate a difference within treatments at *P*< 0.05. Treatments: CT (control), SM5 (5000 mg/L), SM10 (10,000 mg/L), and SM15 (15,000 mg/L). *DAS* days after sowing. Asterisks ***: 0.001, **: 0.01, *: 0.05, *ns* non-significant.

**Fig. 2:**
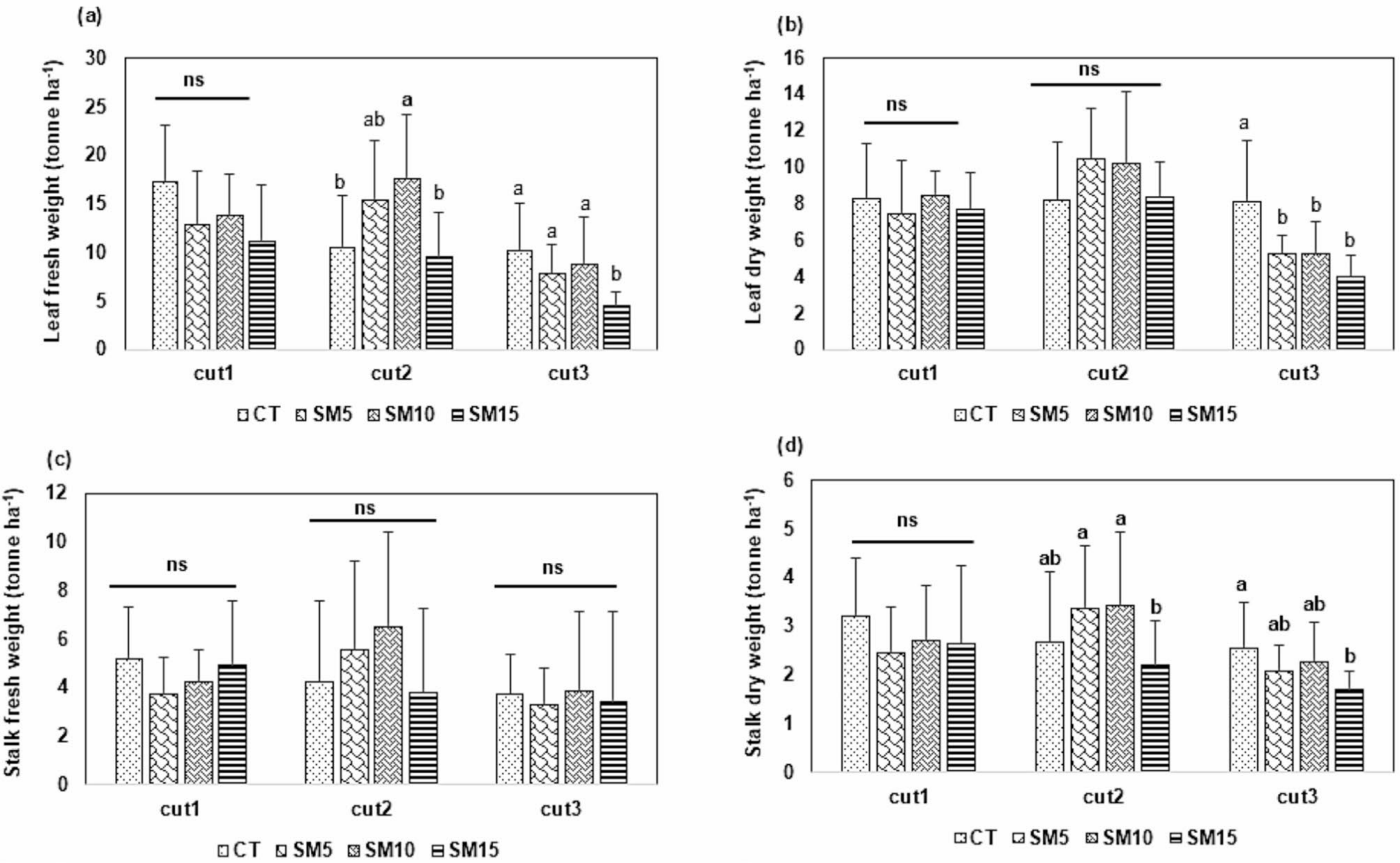
The biomass weight of *Panicum maximum*cv. Mombaça under different salinity treatments. (**a**): Leaf fresh weight; (**b**): Leaf dry weight; (**c**): Stalk fresh weight; (**d**): Stalk dry weight. Data is expressed as mean ± standard deviation (SD). Error bars represent the standard deviation. Different lower superscript letters within each cut indicate a difference amongst treatments at *P* < 0.05, whereas ns means non-significant differences. Treatments: CT (control), SM5 (5000 mg/L), SM10 (10,000 mg/L), and SM15 (15,000 mg/L)

The results of the fresh and dry weight of Mombaça leaves and stalks are presented in Fig. 2. In cut 1, there was a variation in the leaf fresh weight and dry weight among treatments, with CT recording the highest values compared to other treatments. In cut 2, SM5 and SM10 recorded higher values for the leaf fresh weight and dry weight compared to other treatments. In cut 3, however, SM15 significantly (*p* < 0.05) recorded the lowest values for the leaf fresh weight and dry weight compared to other treatments. Similar observations were recorded for the stalk fresh weights and dry weights among treatments across all the cuts.

### Fiber fraction and nutrient composition of leaves

Results on fiber fraction and nutrient composition of leaves are presented in Table 4. The results showed no significant differences in the neutral detergent fiber (NDF), acid detergent fiber (ADF), and acid detergent lignin (ADL) of leaves and stalks between the different treatments at cut 3. In addition, there were no significant differences in the hemicellulose (HEM), cellulose (CEL), and lignin (LIG) content of Mombaça in all the treatments at cut 3. The crude protein (CP) content in the leaves of the control treatment was significantly higher (11.63%) (*P* < 0.05) when compared with that of salinity treatments at cut 3. At the third cut, the fat and fiber content of the leaves did not differ significantly across treatments. Similar results were noted at the second cut, except that SM15 had low values of NDF, ADL, and CEL contents, CT had low NDF and HEM contents, and SM10 had low HEM content. For the first cut, there were no significant differences between the different treatments in all parameters, except for the CEL content, where the CT had the highest values (32.39%) (*P* < 0.05).

**Table 4:** Fiber fraction and nutrient composition of *Panicum maximum* cv. Mombaça cultivated under different salinity treatments and at different cuts

Cut 1 (30 DAS)									
Treatments	NDF (%)	ADF (%)	ADL (%)	HEM (%)	CEL (%)	LIG (%)	CP (%)	Fats (%)	Fiber (%)
CT	60.93^a^ ± 1.04	38.27^a^ ± 1.18	5.88^a^ ± 0.36	22.66^a^ ± 0.15	32.39^a^ ± 0.87	4.14^ab^ ± 0.37	9.43^a^ ± 0.35	2.92^a^ ± 0.17	26.96^a^ ± 1.15
SM5	59.02^ab^ ± 0.43	35.51^ab^ ± 0.58	5.89^a^ ± 0.41	23.51^a^ ± 0.60	29.62^b^ ± 0.33	3.78^b^ ± 0.27	12.93^a^ ± 2.42	3.66^a^ ± 0.45	24.6^ab^ ± 0.67
SM10	56.97^b^ ± 1.27	34.81^b^ ± 1.04	6.18^a^ ± 0.07	22.16^a^ ± 0.27	28.63^b^ ± 0.98	3.97^ab^ ± 0.14	9.37^a^ ± 0.35	3.18^a^ ± 0.02	24.13^b^ ± 0.66
SM15	57.28^b^ ± 0.21	34.11^b^ ± 1.03	5.89^a^ ± 0.30	23.17^a^ ± 0.82	28.23^b^ ± 0.73	4.71^a^ ± 0.14	8.80^a^ ± 0.29	3.41^a^ ± 0.13	23.50^b^ ± 0.54
Cut 2 (60 DAS)									
CT	60.07^b^ ± 0.35	34.74^ab^ ± 0.89	5.33^a^ ± 0.26	23.53^b^ ± 0.71	30.61^a^ ± 0.3	3.77^a^ ± 0.37	13.57^a^ ± 0.65	3.24^ab^ ± 0.36	31.55^a^ ± 2.65
SM5	63.85^a^ ± 1.19	36.81^a^ ± 0.91	5.24^a^ ± 0.22	27.05^a^ ± 0.57	31.58^a^ ± 0.69	3.51^a^ ± 0.30	8.40^b^ ± 0.42	2.64^b^ ± 0.13	33.70^a^ ± 2.95
SM10	60.82^b^ ± 0.51	36.79^a^ ± 0.50	5.86^a^ ± 0.47	24.92^b^ ± 0.28	30.29^a^ ± 0.62	3.52^a^ ± 0.15	9.03^b^ ± 0.43	3.44^a^ ± 0.15	28.23^a^ ± 3.08
SM15	61.29^b^ ± 0.77	32.71^b^ ± 0.52	4.16^b^ ± 0.02	28.27^a^ ± 0.40	28.20^b^ ± 0.65	3.17^a^ ± 0.17	9.67^b^ ± 0.93	3.33^ab^ ± 0.06	30.36^a^ ± 3.30
Cut 3 (90 DAS)									
CT	57.87^a^ ± 0.44	33.45^b^ ± 0.76	4.51^b^ ± 0.29	24.41^a^ ± 0.91	28.94^a^ ± 0.54	3.82^a^ ± 0.37	11.63^a^ ± 0.49	3.09^a^ ± 0.28	32.51^a^ ± 3.24
SM5	58.92^a^ ± 1.15	39.63^a^ ± 0.94	9.17^a^ ± 0.23	19.29^a^ ± 0.5	30.46^a^ ± 0.98	5.78^a^ ± 0.17	8.33^b^ ± 0.41	3.10^a^ ± 0.09	30.29^a^ ± 2.62
SM10	59.97^a^ ± 1.71	36.63^ab^ ± 1.81	7.32^ab^ ± 1.42	23.34^a^ ± 3.04	29.31^a^ ± 0.98	4.64^a^ ± 0.81	8.13^b^ ± 0.49	2.77^ab^ ± 0.08	33.49^a^ ± 0.40
SM15	60.37^a^ ± 2.06	37.01^ab^ ± 1.29	7.56^ab^ ± 1.65	23.36^a^ ± 3.29	29.45^a^ ± 0.92	4.65^a^ ± 1.33	8.50^b^ ± 0.35	2.24^b^ ± 0.35	33.99^a^ ± 0.35
DAS*Treatments	ns	ns	ns	ns	ns	ns	ns	ns	ns

Data are described as means ± standard deviation (SD) (n = 3). Different lower superscript letters in each column per cut represent a difference between treatments at *P *< 0.05. *NDF* Neutral detergent fiber, *ADF* Acid detergent fiber, *ADL* Acid detergent lignin, *HEM* hemicellulose, *CEL* cellulose, *LIG* lignin, and *CP* crude protein. Treatments: CT (control), SM5 (5000 mg/L), SM10 (10,000 mg/L), and SM15 (15,000 mg/L). *DAS* days after sowing. Asterisks ***: 0.001, **: 0.01, *: 0.05, *ns* non-significant

### In-vitro digestibility

Table 5 summarizes the results of in vitro true digestibility (IVTD DM%) of the forage biomass of Mombaça. In cut 1, there was a variation in the in vitro true digestibility of dry matter (IVTD), digestible organic matter (DOM), metabolic energy (ME), short-chain fatty acids (SCFA), total digestible nutrients (TDN), net energy (NE), and microbial protein (MP) across the treatments with SM5 and SM15 significantly recording higher values for DOM, ME, TDN, and MP compared with other treatments. No significant differences were observed in the IVTD, DOM, ME, SCFA, and TDN in the forage biomass among all treatments at cut 2. The NE and MP were significantly higher in the CT and SM15, respectively. However, at cut 3, there were no significant differences between the salinity treatments in all previous parameters except for NE. Moreover, the CT significantly had higher values of all these factors compared with the salinity treatments.

**Table 5:** Results of in vitro digestibility of *Panicum maximum* cv. Mombaça cultivated under different salinity treatments and different cuts

Cut 1 (30 DAS)								
Treatment	IVTD (DM %)	DOM (%)	ME (Mj kg^−1^ DM)	ME (Mcal kg^−1^ DM)	SCFA (mmol ml^−1^ gas)	TDN (%)	NE (Mcal IB ^−1^ )	MP(g kg^−1^ DOM)
CT	36.07^b^ ± 2.26	45.57^b^ ± 0.65	6.71^b^ ± 0.09	1.61^b^ ± 0.02	0.63^b^ ± 0.02	46.28^b^ ± 0.52	3.55^b^ ± 0.02	54.97^b^ ± 0.78
SM5	36.00^b^ ± 0.64	49.89^a^ ± 1.05	7.37^a^ ± 0.16	1.76^a^ ± 0.04	0.71^b^ ± 0.02	49.65^a^ ± 0.83	3.85^a^ ± 0.08	60.18^a^ ± 1.27
SM10	39.68^b^ ± 2.04	45.89^b^ ± 0.86	6.80^b^ ± 0.13	1.62^b^ ± 0.03	0.64^b^ ± 0.02	46.58^b^ ± 0.70	3.56^ab^ ± 0.03	55.35^b^ ± 1.03
SM15	45.98^a^ ± 2.28	51.06^a^ ± 1.82	7.60^a^ ± 0.28	1.82^a^ ± 0.07	0.79^a^ ± 0.05	50.91^a^ ± 1.49	3.70^b^ ± 0.06	61.60^a^ ± 2.19
Cut 2 (60 DAS)								
CT	41.59^a^ ± 2.29	50.36^a^ ± 1.76	7.45^a^ ± 0.27	1.78^a^ ± 0.06	0.71^a^ ± 0.04	50.09^a^ ± 1.44	3.88^a^ ± 0.07	60.4^ab^ ± 1.99
SM5	39.74^a^ ± 3.04	46.91^a^ ± 1.98	6.98^a^ ± 0.30	1.67^a^ ± 0.07	0.68^a^ ± 0.05	47.59^a^ ± 1.59	3.55^c^ ± 0.07	56.59^a^ ± 2.39
SM10	37.06^a^ ± 0.69	48.98^a^ ± 1.09	7.27^a^ ± 0.17	1.74^a^ ± 0.04	0.73^a^ ± 0.03	49.13^a^ ± 0.90	3.64^bc^ ± 0.03	59.08^ab^ ± 1.31
SM15	41.47^a^ ± 1.49	51.45^a^ ± 0.71	7.66^a^ ± 0.12	1.83^a^ ± 0.03	0.79^a^ ± 0.02	51.20^a^ ± 0.65	3.75^ab^ ± 0.02	62.06^a^ ± 0.86
Cut 3 (90 DAS)								
CT	45.87^a^ ± 2.76	52.49^a^ ± 1.71	7.81^a^ ± 0.26	1.87^a^ ± 0.06	0.79^a^ ± 0.05	52.04^a^ ± 1.41	3.87^a^ ± 0.04	63.32^a^ ± 2.06
SM5	27.78^b^ ± 2.14	37.54^b^ ± 0.88	5.50^b^ ± 0.13	1.32^b^ ± 0.03	0.42^b^ ± 0.02	39.71^b^ ± 0.70	3.26^c^ ± 0.03	45.28^b^ ± 1.06
SM10	34.47^b^ ± 2.19	43.19^b^ ± 2.67	6.42^b^ ± 0.41	1.53^b^ ± 0.10	0.57^b^ ± 0.07	44.40^b^ ± 2.17	3.43^bc^ ± 0.08	52.09^b^ ± 3.21
SM15	30.23^b^ ± 1.77	44.19^b^ ± 0.74	6.52^b^ ± 0.11	1.56^b^ ± 0.03	0.59^b^ ± 0.02	45.09^b^ ± 0.60	3.49^b^ ± 0.02	53.30^b^ ± 0.89
DAS*Treatments	ns	ns	ns	ns	ns	ns	ns	ns

Data is represented as mean ± SD (*n* = 3). Different lower superscript letters in each column per cut represent a difference between treatments at *P* < 0.05. *INTD* in vitro true digestibility, *DOM* digestible organic matter, *ME* metabolic energy, *SCFA* short-chain fatty acids, *TDN* total dissolved nutrients, *NE* net energy, and *MP* microbial protein. Treatments: CT (control), SM5 (5000 mg/L), SM10 (10,000 mg/L), and SM15 (15,000 mg/L). *DAS* days after sowing. Asterisks ***: 0.001, **: 0.01, *: 0.05, *ns* non-significant.

### Fish growth performance

The growth performance of striped catfish(*Pangasianodon hypophthalmus*) reared under various salinity treatments is summarized in Table 6. The fish reared in the salinity treatments of SM5 and SM10 showed no significant differences in their final weights. However, their final weights were significantly higher than those in SM15. Furthermore, the body weight gain (BWG) of fish in SM5 and SM10 was significantly (*P* < 0.05) higher than that of SM15. On the other hand, fish reared in SM15 had a significantly (*P* < 0.05)higher value for the feed conversion ratio (FCR) compared with other salinity treatments. However, fish reared in SM5 and SM10 significantly (*P* < 0.05) recorded higher values for the specific growth rate (SGR) and survival percentage compared with those in SM15. For the condition factor (CF), fish reared at SM5 significantly (*P* < 0.05) recorded higher values compared with those in SM15.

**Table 6:** Growth performance parameters of *Pangasianodon hypophthalmus* reared under different salinity treatments

Growth performance	SM5	SM10	SM15
Initial weight (g)	6.12 ± 0.08^a^	6.24 ± 0.16^a^	6.18 ± 0.09^a^
Final weight (g)	112.34 ± 5.67^a^	111.71 ± 7.16^a^	63.40 ± 2.81^b^
BWG (g)	106.22 ± 3.26^a^	105.47 ± 3.17^a^	57.22 ± 32.73^b^
FI (g)	108.98 ± 2.86	109.21 ± 3.47	124.02 ± 2.81
FCR	1.03 ± 0.04^b^	1.04 ± 0.06^b^	2.17 ± 0.18^a^
SGR (%)	1.62 ± 0.07^a^	1.60 ± 0.04^a^	1.29 ± 0.02^b^
CF	1.09 ± 0.19^a^	0.91 ± 0.25^ab^	0.87 ± 0.12^b^
Survival rate (%)	89.00 ± 0.75^a^	91.00 ± 0.98^a^	70.00 ± 0.51^b^

Data is expressed as means ± standard deviation *SD*. Different lower superscript letters within each column indicate a difference among treatments at *P* < 0.05. *BWG* Body weight gain, *FI* Feed intake, *FCR* Feed conversion ratio, *SGR* Specific growth rate, *CF* Condition factor. Treatments: SM5 (5000 mg/L), SM10 (10,000 mg/L), and SM15 (15,000 mg/L). *DAS* days after sowing

### Fish wastewater quality

Table 7 displays the fish wastewater quality parameters under different salinities. As shown in the Table, SM5 significantly recorded the lowest values for ammonia, ammonium, ammonia-nitrogen, nitrite-nitrogen, and nitrate-nitrogen, followed by SM15 and SM10, respectively.

**Table 7:** Water quality parameters of fish wastewater under different salinity treatments

Treatments	NH_3_^+^ (mg/L)	NH_4_^+^ (mg/L)	NH_3_^+^-*N* (mg/L)	NO_2_^−^-*N* (mg/L)	NO_3_^−^-*N* (mg/L)
SM5	0.29^b^ ± 0.15	0.31^b^ ± 0.16	0.24^b^ ± 0.12	1.78^c^ ± 0.32	11.44^c^ ± 0.62
SM10	1.07^a^ ± 0.49	1.13^a^ ± 0.52	0.88^a^ ± 0.40	10.44^a^ ± 0.79	22.90^a^ ± 0.98
SM15	0.85^ab^ ± 0.13	0.9^ab^ ± 0.13	0.72^ab^ ± 0.12	7.78^b^ ± 0.31	21.08^b^ ± 0.10

Data is expressed as mean ± standard deviation *SD*. Different lower superscript letters indicate a difference within treatments at *P* < 0.05. Water quality parameters: NH_3_^+^ (Ammonia), NH_4_^+^(Ammonium), NH_3_^+^–N (Ammonia-Nitrogen), NO_2_^−^–N (Nitrite-Nitrogen), and NO_3_^−^–N (Nitrate-Nitrogen). Treatments: SM5 (5000 mg/L), SM10 (10,000 mg/L), and SM15 (15,000 mg/L).

### Irrigation water productivity

**Fig. 3:**
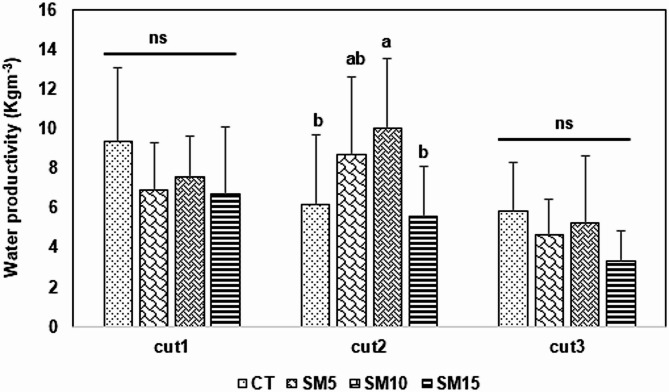
Water productivity of *Panicum maximum*cv. Mombaça under different salinity treatments. Data is expressed as mean ± standard deviation (SD). Error bars represent the standard deviation. Different lower superscript letters within each cut indicate a difference amongst treatments at *P* < 0.05, whereas ns means non-significant differences. Treatments: CT (control), SM5 (5000 mg/L), SM10 (10,000 mg/L), and SM15 (15,000 mg/L)

Water productivity results generally show a negative trend; as the water salinity increases, the water productivity decreases. Comparing the different cuts, cut 1 and cut 3 recorded no significant differences between treatments for the reason being, because during the first cut, there was no salt accumulation in the root zone, and plants can still grow and perform better even at high irrigation water salinities. During the third cut, the plant root stocks are ageing out, there is reduced potential to bring new tillers, and there is a significant accumulation of salts in the root zone. This may justify one of the reasons why the plants generally performed worse in the third cut. The second cut showed interesting results where SM10 outperformed all other treatments, reaching 10.05 Kg/m^3^ of water but without significant differences with SM5. Figure 3

## Discussion

Integrated aquaculture-agriculture systems (IAAS) are correlated with higher agricultural productivity, efficiency, total factor productivity, farm revenue, and returns [43]. The use of fish effluents in irrigated agriculture is gaining popularity in arid and semi-arid areas due to freshwater scarcity [44, 45]. Additionally, the nutrient-rich content of fish effluents reduces the need for chemical fertilizers in soils with poor nutrient levels [45, 46].

In this study, Mombaca was irrigated with saline fish effluents, and the results showed that salinity negatively impacted the morphological plant parameters such as plant height, leaf area, SPAD, and yield compared with freshwater control. Our results are similar to those reported in previous studies on *Sorghum bicolor* [47, 48], *Setaria italic* L [49, 50]., *Panicum virgatum* L [51–54]., and *Lolium perenne* L [55–57]., but different in salinity studies conducted on halophyte plants, *Salicornia neei Lag*., *Apium graveolens* L. and *Paspalum vaginatum* Sw [17]., as well as *Suaeda torreyana* and *Salicornia europaea* [58], cultivated in saline aquaponics systems. Unlike halophytes that can take up salt and show an increase in biomass yield under these forms of IAAS, salt-sensitive plants exhibit a downward trend in yield. This is because salinity stress triggers the accumulation of reactive oxygen species (ROS) in plant tissues, which eventually leads to oxidative damage of nucleic acids, lipids, and proteins, inhibiting plant growth and development [59–61]. High salinity concentrations in the growth media have also been shown to cause reduced water uptake by plant roots and turgor pressure, leading to physiological drought and inhibition of cell elongation, respectively, thus leading to reduced plant growth [62–65]. Furthermore, high salinity leads to the build-up of sodium and chloride ions in plant tissues, which disrupts not only the cellular machinery but also enzymatic activity and nutrient uptake and mobilization, thus leading to the senescence of plant leaves [66, 67]. Likewise, salinity stress has been reported to cause the degradation of chlorophyll molecules, leading to a reduction in the photosynthetic potential of plants, hence leading to a decline in the plant morphological parameters [68–70]. This study also showed a decline in the number of leaves per plant with the increasing number of cuts, especially in the saline treatments. Furthermore, the study results on water productivity suggest that *Panicum maximum* cv. Mombaça can tolerate moderate salinity levels (up to 10,000 mg/L) without significant reductions in water productivity. However, higher salinity levels (15,000 mg/L and above) impair productivity, likely due to osmotic stress, ion toxicity, and reduced water uptake efficiency. Also, as mentioned earlier, salinity reduces photosynthesis, which limits the available energy required for plant growth. Moreover, cutting aggravates this by reducing the photosynthetic area, and as such, the plant will redirect nutrients to maintain essential cellular and metabolic processes rather than producing new leaves [71, 72].

Fiber fraction and in vitro digestibility were used to assess the forage quality of Mombaca cultivated under different salinity treatments and control. The results showed that the neutral detergent fiber (NDF), acid detergent fiber (ADF), acid detergent lignin (ADL), lignin, and fiber percentages generally increased with the increasing number of cuts, especially in extremely saline conditions. Our results are similar to those reported in previous studies [73–76]. The increment in the aforementioned fiber fraction parameters could be due to the development of cell wall constituents with increasing plant maturity [77]. Likewise, lignification of plant tissues increases with plant age [73, 78], and this could explain the increase in the lignin content at cut 3 (90 days after sowing). The crude protein (CP) content ranged from 8.13 to 13.57%, with the control having higher CP levels compared with other treatments. CP is vital in ruminant diets to sustain milk and meat production [79]. The CP content of plants depends on the availability of nitrogen, with higher nitrogen levels leading to higher CP contents [80, 81]. Salinity influences the CP content in forages, and this could be attributed to several factors, such as decreased synthesis of proteins, denaturation of enzymes involved in protein and amino acid biosynthesis, and low availability of amino acids [79, 82]. Nonetheless, the CP contents of Mombaca were within the minimum recommended levels (≥ 7%) for good quality forage [14]. The assessment of in vitro true digestibility (IVTD) and digestible organic matter (DOM) indicated that IVTD and DOM percentages decreased with the increasing number of cuts, especially in extremely saline conditions. As forages mature, their ratio of stems to leaves increases. Since the digestibility of stems is lower than that of leaves (due to high fiber content), this leads to the overall decline in digestibility [73, 83]. Similarly, salinity lowers the digestibility of forages, and this might be due to increased uptake of sodium and chloride ions, which dilute the concentration of proteins, lipids, and carbohydrates. Furthermore, saline conditions lead to the accumulation of ash in plant tissues, which has no nutritional value [84]. Net energy (NE) is one of the parameters used to assess the silage quality of forages. The higher the NE value, the better the silage properties of the forage [85]. This study’s result indicates that better silage properties of Mombaca can still be obtained under salinity concentrations of 15,000 mg/L.

The influence of salinity on fish wastewater quality and fish growth performance was assessed. Higher values for ammonia, ammonium, ammonia-nitrogen, nitrite-nitrogen, and nitrate-nitrogen were observed in SM10. The accumulation of these elements is an indicator of declining rearing water quality as a result of increased amino acid catabolism in fish exposed to salinity stress [14, 86]. Furthermore, high salinity levels have been reported to impede the proliferation and activity of nitrifying bacteria [87, 88]. Even though the ammonia and nitrite levels were above the optimum range required for normal fish growth and survival, we anticipate that their toxicity was reduced due to the increasing concentration of chloride ions (i.e., salinity) in the rearing water. Previous studies have shown that salinity can reduce the nitrite-induced oxidative stress in fish via several mechanisms, such as changes in the expression of certain genes and antioxidant enzyme activity [89, 90]. The body weight gain (BWG) and specific growth rate (SGR) of striped catfish (*Pangasianodon hypophthalmus*) reared at different water salinities were found to be lower in fish grown under extremely saline conditions (15,000 mg/L) compared with other salinities. These findings could be explained by the fact that fish under salinity stress consumed less feed, which led to a poor feed conversion ratio (FCR), poor survival, and specific growth rate consistent with earlier findings of Nguyen et al. [91] and Meritha et al. [92].

## Conclusion

Our study showed that the irrigation of guinea grass *Panicum maximum* cv. Mombaça with aquaculture effluent at salinities of 5000 mg/L, 10,000 mg/L, and 15,000 mg/L does not significantly affect forage dry yield in cut 2. Moderate salinity levels of 5000 mg/L and 10,000 mg/L do not severely affect the forage quality of plants and growth and survival of striped catfish (*Pangasianodon hypophthalmus*), with better forage quality and yield achieved at both cut 1 and cut 2. Consequently, Mombaca can be a substitute for the traditional forage crops for farmers in arid and semi-arid environments, and the integration of striped catfish and Mombaca at water salinities not exceeding 10,000 mg/L could be a feasible alternative for yield diversification in regions of freshwater scarcity and poor nutrient soils. Although the study confirmed the short-term feasibility of using brackish water for Mombaca grass, long-term impacts such as soil salinization remain a concern. Future studies should assess salt accumulation over multiple seasons and explore mitigation strategies like freshwater flushing, crop rotation, and soil amendments. 

## Supplementary Information

Supplementary Material 1.

## Data Availability

The datasets used and/or analyzed during the current study are available from the corresponding author upon reasonable request.
