# Supplementary material for: Assessment of growth performance of striped catfish (Pangasianodon hypophthalmus) and yield of Guinea grass (Panicum maximum cv. Mombaça) under a biosaline integrated aquaculture-agriculture system
Source: BMC Plant Biol. 2025 Sep 22;25:1211. doi: 10.1186/s12870-025-07401-0 (PMC12455846; doi:10.1186/s12870-025-07401-0)
Supplement: Supplementary file 1 — Supplementary Material 1. [file 12870_2025_7401_MOESM1_ESM.docx]

**Supplementary Tables**

**Supplementary Table 1:**Haematological parameters of fish reared under different salinity treatments.

| Treatments | WBC  (µl) | RBC  (µl) | HGB  (%) | HCT  (g/dL) | MCV  (fL) | MCH  (pg) | MCHC  (g/dL) | PLT  (µl) |
| --- | --- | --- | --- | --- | --- | --- | --- | --- |
| SM5 | 19.49^a^ ± 0.335 | 25.6^a^ ± 0.05 | 14.1^a^ ± 0.75 | 40.35^a^ ± 0.09 | 157.6^a^ ± 0.01 | 55.07^a^ ± 2.96 | 34.94^a^ ± 1.88 | 40^a^ ± 5.77 |
| SM10 | 18.72^a^ ± 0.72 | 25.15^a^ ± 0.5 | 13.43^a^ ± 0.75 | 37.45^b^ ± 0.66 | 149.1^c^ ± 0.42 | 53.4^a^ ± 2.66 | 35.87^a^ ± 1.8 | 130^a^ ± 64.3 |
| SM15 | 18.48^a^ ± 0.58 | 21.8^b^ ± 0.23 | 13.13^a^ ± 0.88 | 32.95^c^ ± 0.26 | 151.18^b^ ± 0.4 | 60.25^a^ ± 4.08 | 39.84^a^ ± 2.7 | 43.33^a^ ± 3.33 |

White blood cells (WBC), Red blood cells (RBC), Hemoglobin (HGB), hematocrit (HCT), Mean corpuscular volume (MCV), Mean corpuscular hemoglobin (MCH), Mean corpuscular hemoglobin concentration (MCHC), Platelet count (PLT). Data presented as mean ± SE (n = 3). Different lower superscript letters within every column represent a difference between treatments (*p* < 0.05). Treatments: SM5: 5000 mg/L; SM10: 10,000 mg/L; SM15: 15,000 mg/L.

**Supplementary Table 2:** Blood biochemical parameters of fish reared under different salinity treatments.

| Treatments | Total Protein g/dL | Urea (BUN) mg/dL | Albumin g/dL | Globulin g/dL | Creatinine mg/dL |
| --- | --- | --- | --- | --- | --- |
| SM5 | 4.2^a^ ± 0.18 | 3.55^b^ ± 0.11 | 1.11^a^ ± 0.03 | 3.1^a^ ± 0.16 | 4.17^a^ ± 0.04 |
| SM10 | 3.53^ab^ ± 0.14 | 2.87^b^ ± 0.28 | 1.0^a^ ± 0.01 | 2.53^b^ ± 0.15 | 3.96^a^ ± 0.05 |
| SM15 | 3.32^b^ ± 0.25 | 4.77^a^ ± 0.45 | 1.01^a^ ± 0.11 | 2.31^b^ ± 0.14 | 3.61^a^ ± 0.32 |

Data presented as mean ± SE (n = 3). Different lower superscript letters within every column represent a difference between treatments (p < 0.05). Treatments: SM5: 5000 mg/L; SM10: 10,000 mg/L; SM15: 15,000 mg/L
